# Incidence and Predictors of Synchronous Bone Metastasis in Newly Diagnosed Differentiated Thyroid Cancer: A Real-World Population-Based Study

**DOI:** 10.3389/fsurg.2022.778303

**Published:** 2022-01-24

**Authors:** Lin Qi, Wenchao Zhang, Xiaolei Ren, Ruiling Xu, Chaoqian Liu, Chao Tu, Zhihong Li

**Affiliations:** ^1^Department of Orthopedics, The Second Xiangya Hospital, Central South University, Changsha, China; ^2^Hunan Key Laboratory of Tumor Models and Individualized Medicine, The Second Xiangya Hospital, Changsha, China; ^3^Department of General Surgery, Changhai Hospital, Navy Medical University (Second Military Medical University), Shanghai, China

**Keywords:** synchronous bone metastasis, differentiated thyroid cancer, incidence, prognosis, SEER database

## Abstract

**Background:**

Clinical and sociodemographic characteristics of differentiated thyroid cancer (DTC) patients with synchronous bone metastasis (SBM) remain unclear. This real-world study aimed to elucidate the incidence and prognosis of DTC patients with SBM using population-based data.

**Methods:**

Data of patients with newly diagnosed DTC from 2010 to 2016 were retrieved from the Surveillance, Epidemiology, and End Results (SEER) database. Multivariable logistic regression analysis was utilized to identify predictors of developing SBM in patients with DTC and was further evaluated by receiver operator characteristics (ROC) analysis. Multivariable Cox regression was applied to identify prognostic factors associated with overall survival (OS) and cancer-specific survival (CSS).

**Results:**

A total of 67,176 patients with DTC were screened from the database, with 0.36% (244/67,176) developed SBM. The age-adjusted incidence of SBM in patients with DTC was relatively stable during the study period with an average annual percentage change (AAPC) of 2.52. Multivariable logistic regression analysis recognized seven factors (older age, male gender, black race, other races, follicular histology, the American Joint Committee on Cancer (AJCC) T2, T3, T4 staging, and N1 staging) as predictors of developing SBM among the entire cohort, with the value of area under the curve (AUC) of 0.931 (95% CI: 0.915–0.947). The median survival time of DTC patients with SBM was 22 months (interquartile range, 7–47 months). The multivariable Cox regression analysis indicated multiple metastatic sites, surgical procedures, and chemotherapy as predictors for the survival of patients.

**Conclusions:**

Predictors and prognostic factors of SBM in patients with DTC were identified in this study. Patients with risk factors should be given more attention in clinical practice.

## Introduction

The overall incidence of thyroid cancer has been on the rise in recent decades (1), and it has been reported that the number of patients with new thyroid cancer in 2018 was 567,233 worldwide and thyroid cancer became the sixth most common female cancer in the US (2). Furthermore, estimated new cases of thyroid cancer in the US in 2021 will reach up to 47,200 according to a recent study (3). Differentiated thyroid cancer (DTC) is the most common type of thyroid cancer, which has a good prognosis with 10-year cancer-specific survival (CSS) of more than 70% (4). Distant metastasis in DTC was relatively rare and has been regarded as the poor prognostic factor (5). It has been demonstrated that the 10-year overall survival (OS) rate of DTC patients with pulmonary metastasis was <50%, much lower than that of DTC patients with no distant metastasis (6).

Second to lung, bone was reported to be one of the most common sites for metastasis of DTC, and it was still of great challenge to improve the prognosis in DTC patients with bone metastasis (BM) (7). Spine (34.6%), pelvis (25.5%), sternum and ribs (18.3%), extremities (10.2%), shoulder girdle (5.4%), and craniomaxillofacial (5.4%) were the most common sites of BM in DTC (8). Although BM was associated with a relatively poor prognosis, early detection and appropriate treatment could significantly alleviate suffering of these patients (9). For instance, bone stability of patients could be improved by using percutaneous osteoplasty combined with radioiodine therapy (10). Prompt diagnosis with early initiation of treatment plays a critical role in the outcomes of DTC patients with BM (9, 11, 12).

Therefore, early screening and prediction of BM in DTC were in urgent need for improving the prognosis and reducing the unnecessary costs of patients. Nevertheless, the promising data related to the incidence and predictors of BM in newly diagnosed DTC were extremely limited (9, 13, 14). As most of the relevant studies were case reports with a small sample size, we cannot reconcile these controversial observations to make a conclusion (15–17). Thus, we extracted data from the Surveillance, Epidemiology, and End Results (SEER) database from 2010 to 2016 to conduct this real-world population-based analysis, aiming to identify the trend of incidence and potential predictors of synchronous BM (SBM) in newly diagnosed patients with DTC. Besides, we also aimed to determine the outcome and explore the prognostic factors of DTC patients with SBM.

## Materials and Methods

### Data Source

The Surveillance, Epidemiology, and End Results database was one of the most authoritative cancer databases worldwide, which collected individual data of cancer patients from various regional cancer registries throughout the United States since 1973, covering approximately 30% of the US population (18). Data in the SEER database were deemed reliable ascribe to the rigorous quality control and various data quality assessments. In this study, data of eligible patients were extracted by using the SEER^*^Stat software version 8.3.6 (18). The SEER database provided data of patients up to 2016 based on the November 2018 submission, and data related to specific metastatic sites, such as lung, bone, liver, and brain, were available merely since 2010. Therefore, we could only obtain data of DTC patients with SBM between January 2010 and December 2016.

### Study Design and Participants

**Figure 1:**
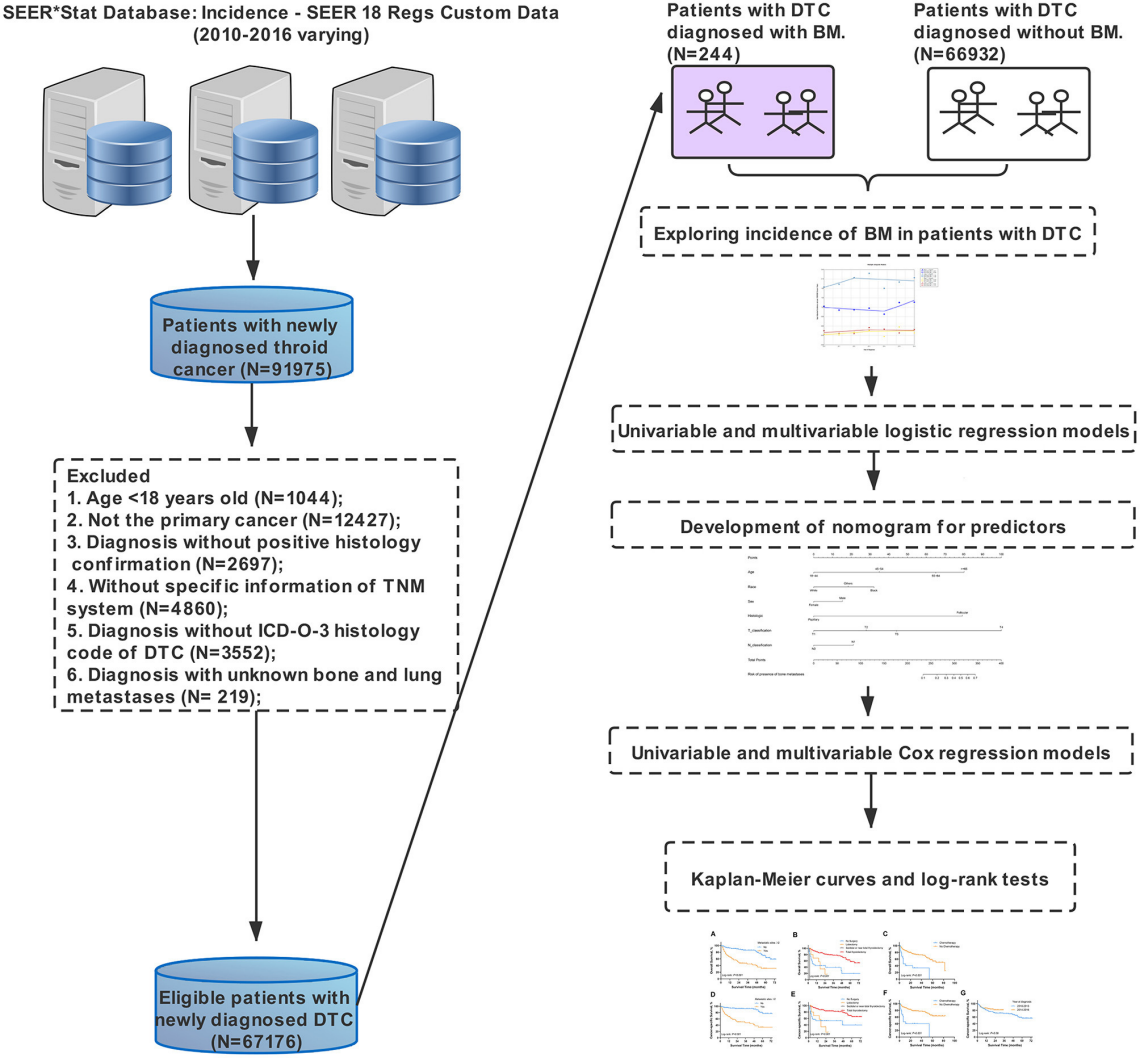
Study design.

Patients with newly diagnosed DTC from 2010 to 2016 were initially enrolled in this study and then screened by following the inclusion criteria below. (1) Age more than 18 years old, (2) diagnosis of DTC with positive histology conformation (the International Classification of Diseases for Oncology, Third Edition [ICD-O-3] histology codes: 8050, 8052, 8260, 8330-8335, and 8340-8344), (3) DTC as the only primary cancer, (4) known survival status and specific time, and (5) known information of SBM. Exclusion criteria included no specific information on the tumor size, tumor node metastasis (TNM) system and unknown SBM information. In this study, variables collected through the SEER database were composed of sociodemographic characteristics (year at diagnosis, age at diagnosis, race, gender, insurance situation, and marital status), clinicopathological characteristics [laterality, histologic type, the American Joint Committee on Cancer (AJCC) clinical stage, AJCC TNM classification, and extraskeletal metastasis], and treatment-related information (surgical procedures, radiotherapy, and chemotherapy). SBM was defined as the diagnosis of BM within 6 months with DTC diagnosis. All data were independently extracted by two researchers. Age was categorized into four groups (≤ 44, 45–54, 55–64, and ≥65) based on the age of onset. The race was classified into white, black, and others (American Indians, Alaska natives, and Asian-Pacific islanders). The year of diagnosis was divided into two periods (2010–2013 and 2014–2016). The primary endpoints in this study were OS and CSS, defined as the time from primary diagnosis to death caused by all reasons and DTC, respectively. The study design with the whole process is shown in Figure 1.

### Statistical Analysis

Description analysis was utilized to present the overall view of frequency and age-adjusted incidence of SBM in newly diagnosed DTC. Frequency was defined as the percentage of DTC patients with SBM among the entire cohort of patients and in the cases who had any distant metastasis. Because this was a real-world population-based study, we principally focused on the entire cohort (patients with newly diagnosed DTC) rather than the subset with metastatic diseases to any sites (lung, liver, and brain). Sociodemographic, clinicopathological, and treatment-related characteristics were introduced to stratify the data of patients who were included. Survival data were demonstrated as Kaplan-Meier survival curves and median with interquartile range. Incidence of newly diagnosed DTC patients with SBM was age adjusted to the 2,000 US standard population and expressed per 1,000,000 person-years. Joinpoint Regression Analysis Program (version 4.7.0.0; National Cancer Institute) was used to calculate annual percentage change (APC) and corresponding 95% CIs.

The differences in sociodemographic, clinicopathological, and treatment characteristics of patients were identified by the Chi-square test and univariable logistic regression analysis. Multivariable logistic regression was used to examine potential predictors of developing SBM in newly diagnosed patients with DTC. The corresponding odds ratios (ORs) along with 95% CI were also obtained. The accuracy of the model was further evaluated by the receiver operating characteristic (ROC) curve analysis. Meanwhile, the nomogram was constructed based on the significant predictors to identify the possibility of SBM in newly diagnosed patients with DTC, by utilizing the R package “rms” (Version 6.2-0). The same procedures were followed to transform a logistic model into the graphical nomogram, which was described in detail previously (19).

Furthermore, univariable and multivariable Cox regression analyses were conducted to identify prognostic factors for OS and CSS in DTC patients with SBM. Potential time-dependent variables were tested to check the proportional hazards assumption, using the “cox.zph” in the R package “survival” (Version 3.2-11). Marital status, surgical procedures, and radiotherapy were treated as time-dependent variables in the Cox regression model (*p* < 0.05) (Supplementary Figures S1, S2). Log-rank tests were conducted for survival data. All statistical analyses were conducted among both the entire cohort and the subset with metastatic diseases, by utilizing SPSS version 24 for Windows (IBM, Armonk, NY, USA). Survival curves were generated by GraphPad Prism 8. The nomogram was established using R version 3.5.3. Two-sided *p* < 0.05 was defined as statistical significance.

## Results

### Characteristics of Patients

A total of 67,176 patients were diagnosed with DTC in the United States from 2010 to 2016 and met the inclusion criteria. Within the entire cohort, 702 patients (1.045%) were diagnosed with distant metastasis to any site, i.e., 323 men (46.01%) and 379 women (53.99%). SBM accounts for 0.36% (244/67,176) among the entire group and 34.76% (244/702) among the subset with all distant metastasis in patient with DTC. The sociodemographic, clinicopathological, and treatment-related characteristics of the population in this study are present in Table 1. The median survival time of DTC patients with SBM was 22 months (interquartile range, 7–47 months).

**Table 1:** Demographic and clinical characteristics of DTC patients with SBM.

**Variable**		**Patients, No**.		**Proportion of BM, %**		**Survival among patients with SBM, median (IQR), mo**
	**With DTC** **(*n =* 67176)**	**With metastatic disease** **(*n =* 702)**	**With SBM** **(*n =* 244)**	**Among entire cohort**	**Among subset with metastatic disease**	
**Year at diagnosis**						
2010–2013	37542	367	122	0.32	33.24	47.00 (14.00–58.00)
2014–2016	29634	335	122	0.41	36.42	14.00 (5.00–23.25)
**Age at diagnosis, Years**						
18–44	27185	128	24	0.09	18.75	22.50 (10.25–47.00)
45–54	16284	112	35	0.21	31.25	33.00 (14.00–53.00)
55–64	13432	175	74	0.55	42.29	20.50 (6.00–39.50)
≥65	10275	287	111	1.08	38.68	19.00 (6.00–42.00)
**Race**						
White	53664	521	164	0.31	31.48	22.00 (7.00–47.00)
Black	4685	61	35	0.75	57.38	23.00 (5.00–35.00)
Others^†^	8827	120	45	0.51	37.5	19.00 (6.00–46.50)
**Gender**						
Male	15085	323	103	0.68	31.89	22.00 (9.00–48.00)
Female	52091	379	141	0.27	37.2	21.00 (6.00–43.00)
**Insurance situation**						
Insured	65472	674	236	0.36	35.01	21.50 (7.00–47.00)
Uninsured	1704	28	8	0.47	28.57	33.00 (4.25–46.75)
**Marital status**						
Married	40859	381	138	0.34	36.22	22.00 (9.00–48.00)
Unmarried^‡^	22346	295	99	0.44	33.56	21.00 (5.00–44.00)
Unknown	3971	26	7	0.17	26.92	17.00 (17.00–35.00)
**Laterality**						
Unilateral	66640	699	243	0.36	34.76	22.00 (7.00–47.00)
Bilateral	536	3	1	0.19	33.33	22.00 (7.00–46.50)
**Histologic type**						
Papillary	63945	582	161	0.25	27.66	21.00 (7.00–41.50)
Follicular	3231	120	83	2.57	69.17	23.00 (7.00–49.00)
**AJCC clinical stage^§^**						
I	49301	0	0	0	NA	NA
II	4696	0	0	0	NA	NA
III	9021	0	0	0	NA	NA
IV	4158	702	244	5.87	34.76	22.00 (7.00–47.00)
**AJCC T classification** ^**§**^						
T1	40294	101	44	0.11	43.56	25.50 (6.50–40.25)
T2	11168	82	42	0.38	51.22	26.50 (9.50–51.50)
T3	13838	263	91	0.66	34.6	26.00 (10.00–53.00)
T4	1876	256	67	3.57	26.17	11.00 (5.00–24.00)
**AJCC N classification** ^**§**^						
N0	51387	277	154	0.3	55.6	23.50 (8.00–50.25)
N1	15789	425	90	0.57	21.18	18.50 (6.00–33.00)
**AJCC M classification** ^**§**^						
M0	66474	0	0	0	NA	NA
M1	702	702	244	34.76	34.76	22.00 (7.00–47.00)
**Multifocality**						
No	38843	337	128	0.33	37.98	19.50 (6.00–43.50)
Yes	28333	365	116	0.41	31.78	22.00 (8.50–47.00)
**Extraskeletal metastases to lung, liver and brain, No**.						
0	66734	260	141	0.21	54.23	28.00 (14.00–53.00)
1	82	82	82	100	100	15.00 (6.00–29.50)
2	36	36	21	100	58.33	6.00 (4.00–8.50)
3	1	1	0	100	0	NA
Unknown	323	323	0	82.25	0	NA
**Surgery**						
No	568	87	38	6.69	43.68	6.00 (2.75–22.50)
Lobectomy	8904	33	10	0.11	30.3	16.00 (4.00–23.25)
Subtotal or near–total thyroidectomy	1758	18	1	0.06	5.56	NA
Total thyroidectomy	55946	564	195	0.35	34.57	25.00 (10.00–50.00)
**Radiation therapy**						
No	36712	171	43	0.11	25.15	7.00 (3.00–41.00)
Radiation beam or radioactive implants	1070	163	88	8.22	53.99	11.00 (5.00–30.25)
Radioisotopes	29293	348	97	0.33	27.87	27.00 (17.00–54.00)
Combination^¶^	100	20	16	16	80	35.00 (20.50–59.00)
**Chemotherapy**						
No	66990	638	224	0.33	35.11	22.00 (8.00–47.00)
Yes	186	64	20	10.75	31.25	7.00 (3.25–24.00)

*DTC, Differentiated Thyroid Carcinoma, SBM, Synchronous Bone Metastases, CI, Confidence Interval, IQR, Interquartile Range, NA, Not Applicable.*

† *Including American Indians, Alaska Natives and Asian-Pacific Islanders.*

‡ *Divorced, separated, single (never married), and widowed.*

§ *According to the seventh edition of the AJCC Cancer Staging manual.*

¶ *Combination of beam with implants or isotopes*.

**Figure 2:**
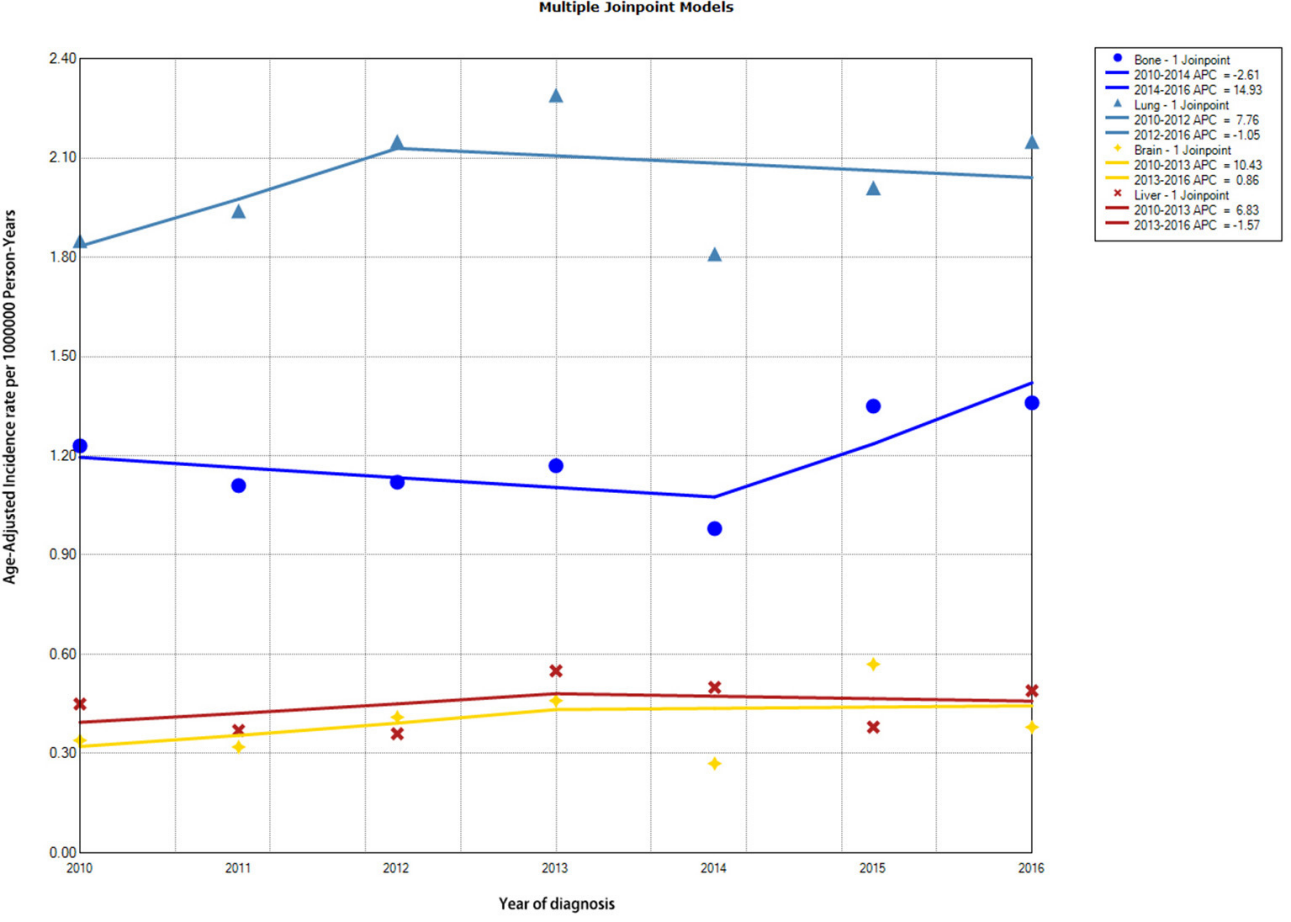
Trends in annual incidence rates of DTC with different distant metastasis to specific sites. DTC, differentiated thyroid cancer.

Incidence trends of DTC patients with specific distant metastasis are demonstrated in Figure 2 and Supplementary Table S1. Incidence rates of SBM in patients with DTC remained relatively stable during the study period (from 1.23 [95% CI, 0.91–1.62] per 1,000,000 person-years in 2010 to 1.36 [95% CI, 1.06–1.74 per 1,000,000 person-years in 2016), with an average annual percentage change (AAPC) of 2.52 (95% CI, −2.81 to 8.14, *p* = 0.28). Overall incidence rates of other specific distant metastasis, such as sites to lung, brain, and liver, were also relatively stable in the study period.

### Predictors of SBM in Patients With DTC

In univariable analysis, age at diagnosis, race, gender, marital status, histology, AJCC T classification, and AJCC N classification showed significant differences among the entire cohort (Supplementary Table S2).

All these statistically significant factors were entered into the multivariable logistic regression analysis, which is shown in Table 2. Age 45–54 years (OR: 2.674, 95% CI: 1.583–4.517, *p* < 0.001), age 55–64 years (OR: 6.090, 95% CI: 3.810–9.734, *p* < 0.001), age ≥65 years (OR: 9.088, 95% CI: 5.771–14.311, *p* < 0.001), black race (OR: 2.348, 95% CI: 1.589–3.470, *p* < 0.001), other races (OR: 1.678, 95% CI: 1.194–2.360, *p* = 0.003), follicular histology (OR: 8.735, 95% CI: 6.336–12.042, *p* < 0.001), AJCC T2 staging (OR: 2.134, 95% CI: 1.367–3.331, *p* = 0.001), AJCC T3 staging (OR: 3.312, 95% CI: 2.250–4.874, *p* < 0.001), AJCC T4 staging (OR: 15.068, 95% CI: 9.748–23.291, *p* < 0.001), and AJCC N1 staging (OR: 1.781, 95% CI: 1.281–2.477, *p* = −0.001) were associated with higher odds of developing SBM in newly diagnosed DTC. Meanwhile, the female gender (OR: 0.632, 95% CI: 0.482–0.828, *p* = 0.001) was significantly associated with a lower possibility of developing SBM in newly diagnosed patients with DTC.

**Table 2:** Multivariable logistic regression for developing SBM in DTC patients.

**Variable**	**Among entire cohort**		**Among subset with metastatic disease**	
	**OR (95% CI)**	***p*-value**	**OR (95% CI)**	***p*-value**
**Age at diagnosis, Years**				
18–44	Reference	NA	Reference	NA
45–54	2.674 (1.583–4.517)	<0.001	1.930 (1.014–3.672)	0.045
55–64	6.090 (3.810–9.734)	<0.001	2.393 (1.334–4.293)	0.003
≥65	9.088 (5.771–14.311)	<0.001	1.800 (1.026–3.159)	0.040
Race				
White	Reference	NA	Reference	NA
Black	2.348 (1.589–3.470)	<0.001	1.859 (1.014–3.407)	0.045
Others^†^	1.678 (1.194–2.360)	0.003	1.037 (0.657–1.639)	0.875
**Gender**				
Male	Reference	NA	NA	NA
Female	0.632 (0.482–0.828)	0.001	NA	NA
**Marital status**				
Married	Reference	NA	NA	NA
Unmarried^‡^	1.239 (0.942–1.629)	0.125	NA	NA
Unknown	0.522 (0.241–1.129)	0.099	NA	NA
**Histologic**				
Papillary	Reference	NA	Reference	NA
Follicular	8.735 (6.336–12.042)	<0.001	3.515 (2.194–5.630)	<0.001
**AJCC T classification^§^**				
T1	Reference	NA	Reference	NA
T2	2.134 (1.367–3.331)	0.001	1.252 (0.651–2.405)	0.500
T3	3.312 (2.250–4.874)	<0.001	0.814 (0.482–1.376)	0.442
T4	15.068 (9.748–23.291)	<0.001	0.711 (0.412–1.225)	0.219
**AJCC N classification^§^**				
N0	Reference	NA	Reference	NA
N1	1.781 (1.281–2.477)	0.001	0.378 (0.257–0.557)	<0.001

*DTC, Differentiated Thyroid Carcinoma, SBM, Synchronous Bone Metastases, OR, odds ratio, CI, Confidence Interval, NA, Not Applicable.*

† *Including American Indians, Alaska Natives and Asian-Pacific Islanders.*

‡ *Divorced, separated, single (never married), and widowed.*

§ *According to the seventh edition of the AJCC Cancer Staging manual*.

**Figure 3:**
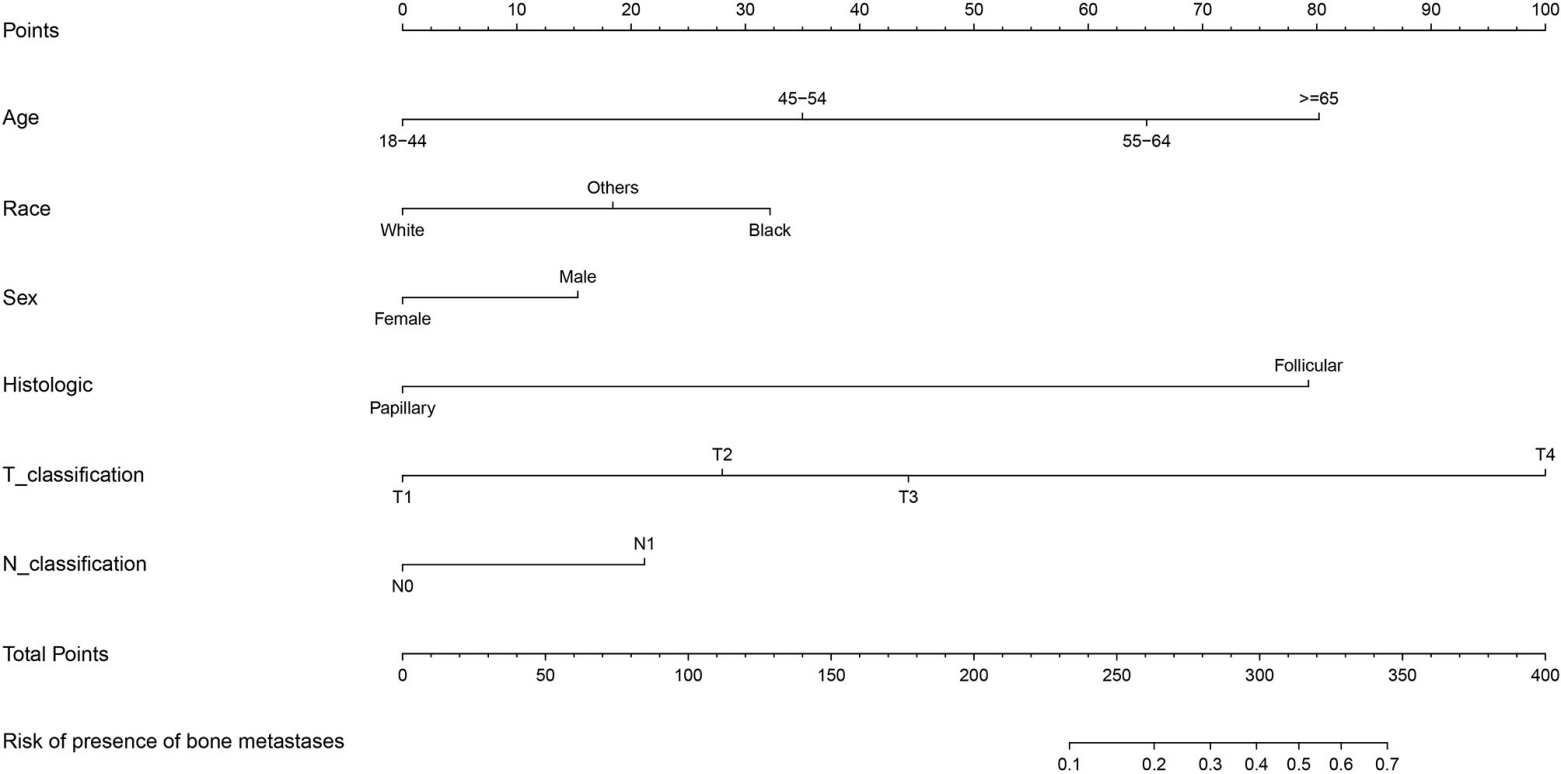
Nomogram for predictors of the presence of SBM in the patients with DTC. SBM, synchronous bone metastasis; DTC, differentiated thyroid cancer.

Moreover, ROC analysis was conducted to further assess the multivariable logistic regression models with significant predictors above. The multivariable logistic regression model of the entire cohort exhibited optimal performance with the value of area under the curve (AUC) of 0.931 (95% CI: 0.915–0.947; Supplementary Figure S3). Furthermore, the nomogram predicting the probability of developing SBM in patients with DTC was further established based on significant predictors identified through multivariable logistic regression analysis (Figure 3). Each variable had a corresponding score in the nomogram (Supplementary Table S3). The probability of developing SBM in patients with DTC could be calculated utilizing this nomogram.

### Survival Analysis

**Figure 4:**
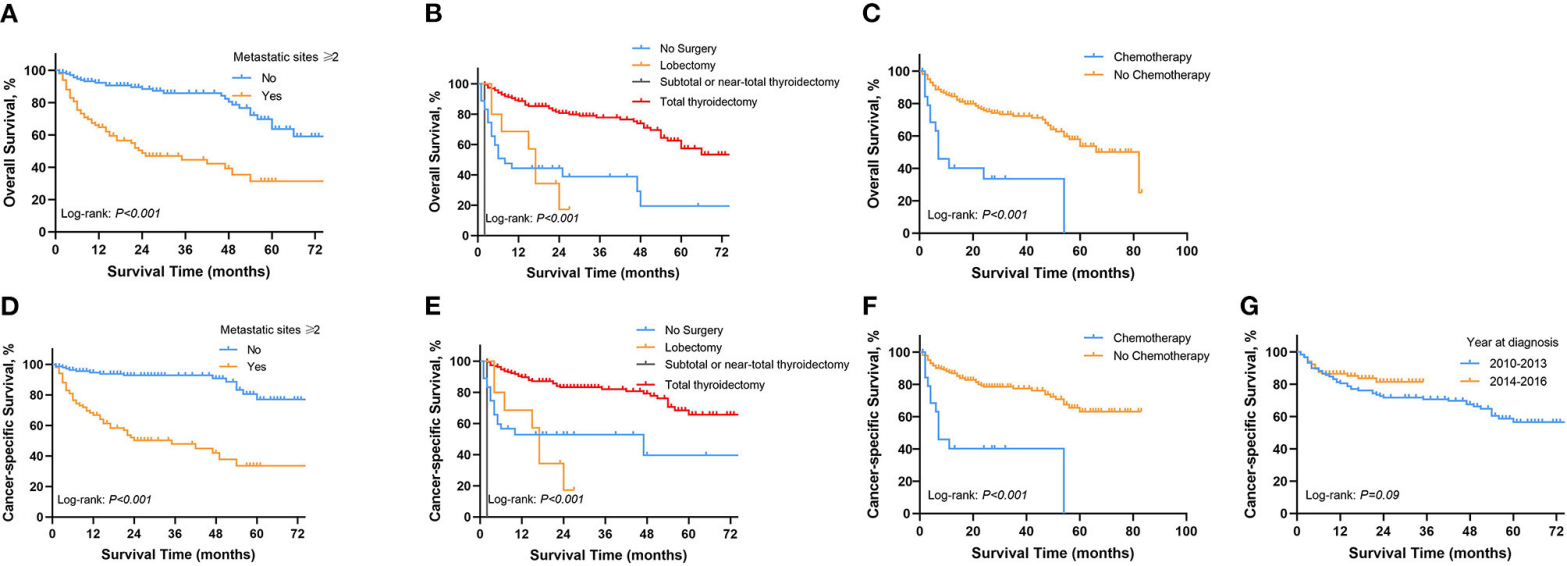
Kaplan-Meier survival curves presenting OS and CSS in Patients with DTC diagnosed with SBM. **(A)** OS between metastasis sites ≥2 and <2. **(B)** OS in different surgical procedures. **(C)** OS of patients with and without chemotherapy. **(D)** CSS between metastasis sites ≥2 and <2. **(E)** CSS in different surgical procedures. **(F)** CSS of patients with and without chemotherapy. **(G)** CSS in different periods of diagnosis. OS, overall survival; CSS, cancer-specific survival; DTC, differentiated thyroid cancer; SBM, synchronous bone metastasis.

In order to explore prognostic factors in DTC patients with SBM, we conducted following survival analysis. The univariable Cox regression analysis for OS and CSS in DTC patients with SBM is shown in Supplementary Table S4. On multivariable Cox regression analysis with time-dependent covariates among the cohort of DTC patients with SBM (Table 3), we identified three significant prognostic factors that were significantly associated with OS, such as metastatic sites ≥2 (hazard ratio [HR]: 3.077, 95% CI: 1.820–5.205, *p* < 0.001), lobectomy (HR: 1.067, 95% CI: 1.001–1.138, *p* = 0.048), subtotal or near-total thyroidectomy (HR: 3.404, 95% CI: 1.051–11.024, *p* = 0.041), and chemotherapy (HR: 2.463, 95% CI: 1.276–4.755, *p* < 0.001). Moreover, prognostic factors associated with CSS of DTC patients with SBM were diagnosis at 2014–2016 (HR: 0.569, 95% CI: 0.305–0.987, *p* = 0.049), metastatic sites ≥2 (HR: 5.077, 95% CI: 2.695–9.566, *p* < 0.001), lobectomy (HR: 1.083, 95% CI: 1.007–1.166, *p* = 0.032, and chemotherapy (HR: 2.788, 95% CI: 1.374–5.657, *p* = 0.005). OS of DTC patients with SBM estimates as stratified by metastatic sites (Figure 4A), surgery (Figure 4B), and chemotherapy (Figure 4C), and CSS estimates as stratified by metastatic sites (Figure 4D), surgery (Figure 4E), chemotherapy (Figure 4F), and year at diagnosis (Figure 4G) are illustrated in Figure 4.

**Table 3:** Multivariable analysis for OS and CSS in patients with DTC diagnosed with SBM.

**Variable**	**Overall survival**		**Cancer-specific survival**	
	**HR (95% CI)**	***p*-value**	**HR (95% CI)**	***p*-value**
**Year at diagnosis**				
2010–2013	NA	NA	Reference	NA
2014–2016	NA	NA	0.569 (0.305–0.987)	0.049
**AJCC T classification^§^**				
T1	Reference	NA	Reference	NA
T2	1.029 (0.413–2.567)	0.951	0.897 (0.312–2.578)	0.840
T3	0.803 (0.361–1.788)	0.592	0.643 (0.260–1.588)	0.339
T4	1.446 (0.619–3.375)	0.394	1.004 (0.383–2.629)	0.994
**AJCC N classification^§^**				
N0	Reference	NA	Reference	NA
N1	1.349 (0.822–2.214)	0.236	1.547 (0.887–2.698)	0.124
**Metastatic sites≥2**				
No	Reference	NA	Reference	NA
Yes	3.077 (1.820–5.205)	<0.001	5.077 (2.695–9.566)	<0.001
**Surgery**				
No	Reference	NA	Reference	NA
Lobectomy	1.067 (1.001–1.138)	0.048	1.083 (1.007–1.166)	0.032
Subtotal or near-total thyroidectomy	3.404 (1.051–11.024)	0.041	2.734 (0.832–8.989)	0.098
Total thyroidectomy	0.977 (0.952–1.002)	0.066	0.986 (0.947–1.027)	0.506
**Chemotherapy**				
No	Reference	NA	Reference	NA
Yes	2.463 (1.276–4.755)	<0.001	2.788 (1.374–5.657)	0.005

*DTC, Differentiated Thyroid Carcinoma, SBM, Synchronous Bone Metastases, HR, hazard ratio, CI, Confidence Interval, NA, Not Applicable.*

§ *According to the seventh edition of the AJCC Cancer Staging manual*.

## Discussion

The incidence of BM in patients with DTC was relatively lower compared with that in other malignant cancers, such as lung cancer and breast cancer (20, 21). Besides, BM often occurs in medullary and undifferentiated thyroid cancer (22), and limited data about BM in patients with DTC have been reported. Despite several case reports and cohort studies (23–25), little was known about its incidence, risk factors, and prognosis in DTC. In this real-world population-based study, we analyzed the trend in incidence, predictors, and prognosis of SBM in newly diagnosed patients with DTC, aiming to promote the understanding of the current situation and the association between clinical pattern and SBM. To the best of our knowledge, this study included the largest sample size of DTC patients with SBM compared with previous ones.

In the present study utilizing the SEER database, SBM accounts for 0.36% (244/67,176) among the entire group and 34.76% (244/702) among the subset with metastatic diseases in newly diagnosed patients with DTC, which was lower than the proportion (2.0%) in the study of Yorihisa et al. (9) and the proportion (5.51%) in the study of Marie-odile et al. (26). The sample size in these two studies was relatively small and they only included patients in a single institution, so the results in the above studies were not representative enough. In addition, the above two studies were conducted with date in the early period, while the current study focused on patients diagnosed from 2010 to 2016. With the development of screening methods, early detection of asymptomatic thyroid cancer and timely interventions, such as surgery, have substantially enhanced the prognosis of patients with thyroid cancer. Therefore, the frequency of bone metastases in patients with DTC may have reduced compared with that in the early decades. The incidence of thyroid cancer was increasing continuously from 1974 to 2013 in the United States (1). To explore the trend of incidence of SBM in patients with DTC, Jointpoint regression analysis was introduced, and the result indicated that the age-adjusted incidence of SBM in patients with DTC was relatively stable during the study period, which was not reported before.

By using multivariable logistic regression analysis, predictors of developing SBM in newly diagnosed DTC were identified. Older age, black race, male gender, follicular histology, and more advanced AJCC T stage and N stage were significantly associated with of presence of BM in patients with DTC. In a meta-analysis that enrolled 34 articles with 73,219 patients with DTC, age ≥45 years, male gender, and follicular histology were also demonstrated to be significant predictors of distant metastasis, which was consistent with the current analysis (27). Follicular thyroid cancer was characterized with higher invasion and distant metastasis compared with papillary thyroid cancers, and the rate of BM was relatively higher correspondently (28). According to a population analysis, i.e., patients with DTC from 1988 to 2009 in the SEER database, patients with distant metastasis tended to have received radiation therapy rather than surgery (29). However, as it was discussed, the SEER database did not provide information about specific anatomic sites of distant metastasis at that time, so the detailed data for BM were not available in that analysis.

Furthermore, the optimal performance of the multivariable logistic regression model in the entire cohort was authenticated by ROC analysis, in which the AUC value was 0.931 (95% CI: 0.915–0.947). Besides, the nomogram established in this study is an efficient tool to help clinical workers' decision-making owing to its user-friendly interface and optimal predictive ability. Similarly, the nomograms based on logistic regression were used to visually interpret the prediction model in several studies (19, 30, 31). However, the present predictive model lacked internal and external validations due to the limited sample size. It is still noteworthy that the nomogram should be applied with caution. Nevertheless, we were not capable of figuring out the specific common sites of BM because there was no relevant data documented in the SEER database until now.

Prognostic factors for OS and CSS of DTC patient with BM were analyzed using the multivariable Cox regression analysis. Results revealed that patients with multiple metastatic sites have higher HR for OS and CSS, which was in coherence with previous studies (11, 32). Surgery was the mainstay of therapy for thyroid cancer patients, especially in patients without distant metastasis (33). Surgical options include thyroid total thyroidectomy or lobectomy, and it was still in controversy on how to choose optimal surgical procedure because of the difficulty in balancing surgical effect and complication (34). Total thyroidectomy reduces recurrence and allows early detection of recurrence using ultrasonography compared with lobectomy, thereafter improving the prognosis compared with other treatment options. The most used radioisotope in DTC treatment was radioiodine, while the application was still of wide divergence, partially due to the lack of evidence concerning prospective randomized controlled trials (35). Patients with BM could benefit from radioiodine in both suppressing the progression of tumor and reducing recurrence of primary cancer. It was reported in some retrospective studies that the application of radioiodine could improve the survival of patients with DTC and that of patients with BM (9, 36–38). Especially, radioiodine therapy was effective in the ablation of thyroid remnant after surgery (39). Recently, a retrospective study that enrolled 74 thyroid cancer patients with BM has demonstrated that external beam radiation therapy (EBRT) significantly increased the prognosis (40). Nevertheless, EBRT was reported to have acute toxicities, such as esophageal stricture, so it was used under relatively strict indications (41). Besides, combining radioiodine and external beam radiation therapy seems advantageous in some cases (42). However, the treatment effect of total thyroidectomy and radioiodine in DTC patients with BM needs to be further explored in the future study. It was reported that older age, positive lymph node, and black race were also significant predictors of prognosis of thyroid cancer patients with BM (24, 25). Further study is necessary to shed light on potential prognostic factors.

Although this was a real-world population-based study that enrolled the largest cohort of DTC patients with SBM, limitations in this study were unneglectable. Because it was a retrospective study based on current database records, some specific parameters were unavailable. Firstly, the further detailed information of SBM, such as specific sites of bone, was not available in the SEER database, which limited further analysis. Secondly, asynchronous BM was not recorded in the database so we only had information on synchronous metastasis, probably making the incidence underestimated. Thirdly, more detailed information about radiation or chemotherapy, such as chemotherapy regimens, doses, and the specific number of cycles, was not recorded in the SEER database. Fourthly, except for the survival data, other outcomes, such as complications of exposures or interventions, were not reported. We could only assess the prognosis by OS and CS. Another limitation of the study was the absence of internal and external validations due to the limited sample size. Finally, data on specific metastatic sites, such as lung, bone, liver, and brain, were available in the SEER database only since 2010, leading to some inevitable bias. Therefore, more convincible clinical studies especially the randomized clinical trials were necessitated in the future.

In summary, the results of this real-world population-based study highlighted the incidence, predictors, and prognostic factors of SBM in newly diagnosed patients with DTC. The age-adjusted incidence of SBM in DTC was relatively stable during the study period. Older age, male gender, black race, other races, follicular histology, more advanced T staging, and N staging were significant predictors for developing SBM in patients with DTC. Thus, appropriate screenings for patients with these risk factors are recommended. Moreover, in DTC patients with SBM, multiple metastatic sites could serve as the indicator of poor prognosis, which provided evidence for future clinical practice.

## Data Availability Statement

The original contributions presented in the study are included in the article/Supplementary Material, further inquiries can be directed to the corresponding author/s.

## Author Contributions

ZL and CT designed the study and made final approval of the version. LQ performed study concept and design and wrote the manuscript. XR, CL, RX, and WZ helped with data analysis. LQ interpreted results and helped to write the manuscript. All authors contributed to the article and approved the submitted version.

## Funding

This study was supported by the National Natural Science Foundation of China (Nos. 81902745, 82172500, and 82103228), Hunan Provincial Research and Development Program in Key Areas (2019WK2071), Hunan Provincial Innovation Foundation for Post-graduate (CX20190160), and China Postdoctoral Science Foundation (2021M693557).

## Conflict of Interest

The authors declare that the research was conducted in the absence of any commercial or financial relationships that could be construed as a potential conflict of interest.

## Publisher's Note

All claims expressed in this article are solely those of the authors and do not necessarily represent those of their affiliated organizations, or those of the publisher, the editors and the reviewers. Any product that may be evaluated in this article, or claim that may be made by its manufacturer, is not guaranteed or endorsed by the publisher.

## Supplementary Material

Supplementary Figure S1 Schoenfeld residuals for the variables with OS by the time. OS, overall survival.

Supplementary Figure S2 Schoenfeld residuals for the variables with CSS by the time. CSS, cancer-specific survival.

Supplementary Figure S3 ROC curve of the final multivariable logistic regression model. **(A)** Among the entire cohort. **(B)** Among subset with metastatic diseases. ROC, receiver operator characteristics.

Supplementary Table S1 Trends in age-adjusted incidence of distant metastases with DTC from 2010 to 2016.

Supplementary Table S2 Univariable logistic regression of developing SBM in DTC patients.

Supplementary Table S3 Detailed scores of predictors in the nomograms.

Supplementary Table S4 Univariable analysis for OS and CSS in patients with DTC diagnosed with SBM.

## Abbreviations

DM: distant metastasis
DTC: differentiated thyroid cancer
SBM: synchronous bone metastasis
SEER, Surveillance, Epidemiology: and End Results
ROC: receiver operating characteristic
APCC: average annual percentage change
AUC: value of area under curve
AJCC: American joint committee on cancer
APC: annual percentage change
OR: odds ratio
CI: confidence interval
EBRT: external beam radiation therapy
OS: overall survival
CSS: cancer-specific survival.
