# Supplementary material for: Incidence and Predictors of Synchronous Bone Metastasis in Newly Diagnosed Differentiated Thyroid Cancer: A Real-World Population-Based Study
Source: Front Surg. 2022 Jan 24;9:778303. doi: 10.3389/fsurg.2022.778303 (PMC8819693; doi:10.3389/fsurg.2022.778303)
Supplement: Supplementary Table S1 — Trends in age-adjusted incidence of distant metastases with DTC from 2010 to 2016. [file Table_1.DOCX]

| Supplemental table 1. Trends in age-adjusted incidence of distant metastases with DTC from 2010 to 2016 | | | | | | | | | |
| --- | --- | --- | --- | --- | --- | --- | --- | --- | --- |
|  | Overall |  | Trend 1 |  |  | Trend 2 |  |  |  |
|  | Mean APC (95%CI) | p Value | Year | APC (95%CI) | p Value | Year | APC (95%CI) | p Value |  |
| Sites |  |  |  |  |  |  |  |  |  |
| Bone | 2.52 (-2.81 to 8.14) | 0.28 | 2010-2014 | -2.61 (-12.12 to 7.93) | 0.38 | 2014-2016 | 14.93 (-13.15 to 52.08) | 0.17 |  |
| Lung | 1.23 (-3.13 to 5.78) | 0.51 | 2010-2012 | 7.76 (-51.45 to 139.21) | 0.73 | 2012-2016 | -1.05 (-20.74 to 23.52) | 0.86 |  |
| Brain | 5.27 (-6.78 to 18.88) | 0.33 | 2010-2013 | 10.43 (-64.65 to 245.03) | 0.74 | 2013-2016 | 0.86 (-71.06 to 251.51) | 0.98 |  |
| Liver | 2.46 (-5.72 to 11.36) | 0.49 | 2010-2013 | 6.83 (-32.97 to 70.25) | 0.60 | 2013-2016 | -1.57 (-35.25 to 49.62) | 0.89 |  |

DTC, Differentiated Thyroid Carcinoma, APC, annual percent change
