# Supplementary material for: Incidence and Predictors of Synchronous Bone Metastasis in Newly Diagnosed Differentiated Thyroid Cancer: A Real-World Population-Based Study
Source: Front Surg. 2022 Jan 24;9:778303. doi: 10.3389/fsurg.2022.778303 (PMC8819693; doi:10.3389/fsurg.2022.778303)
Supplement: Supplementary Table S2 — Univariable logistic regression of developing SBM in DTC patients. [file Table_2.DOCX]

Supplemental Table 2 Univariable Logistic Regression of Developing SBM in DTC Patients

| Variable | Among entire cohort | | Among subset with  metastatic disease | |
| --- | --- | --- | --- | --- |
|  | OR (95% CI) | p Value | OR (95% CI) | p Value |
| Age at diagnosis, Years |  |  |  |  |
| 18-44 | Reference | NA | Reference | NA |
| 45-54 | 2.438 (1.450-4.099) | 0.001 | 1.970 (1.084-3.579) | 0.026 |
| 55-64 | 6.269 (3.954-9.940) | <0.001 | 3.175 (1.858-5.425) | <0.001 |
| ≥65 | 12.359 (7.945-19.225) | <0.001 | 2.733 (1.652-4.521) | <0.001 |
| Race |  |  |  |  |
| White | Reference | NA | Reference | NA |
| Black | 2.455 (1.703-3.541) | <0.001 | 2.930 (1.708-5.029) | <0.001 |
| Others† | 1.672 (1.201-2.327) | 0.002 | 1.306 (0.864-1.974) | 0.205 |
| Gender |  |  |  |  |
| Male | Reference | NA | Reference | NA |
| Female | 0.395 (0.306-0.509) | <0.001 | 1.265 (0.925-1.731) | 0.141 |
| Insurance situation |  |  |  |  |
| Insured | Reference | NA | Reference | NA |
| Uninsured | 1.304 (0.643-2.642) | 0.461 | 0.742 (0.322-1.711) | 0.484 |
| Marital status |  |  |  |  |
| Married | Reference | NA | Reference | NA |
| Unmarried‡ | 1.313 (1.014-1.701) | 0.039 | 0.889 (0.646-1.224) | 0.472 |
| Unknown | 0.521 (0.244-1.114) | 0.093 | 0.649 (0.266-1.582) | 0.341 |
| Laterality |  |  |  |  |
| Unilateral | Reference | NA | Reference | NA |
| Bilateral | 0.511 (0.072-3.647) | 0.503 | 0.938 (0.085-10.400) | 0.959 |
| Histologic type |  |  |  |  |
| Papillary | Reference | NA | Reference | NA |
| Follicular | 10.446 (7.996-13.646) | <0.001 | 5.866 (3.824-8.998) | <0.001 |
| AJCC T classification§ |  |  |  |  |
| T1 | Reference | NA | Reference | NA |
| T2 | 3.453 (2.261-5.273) | <0.001 | 1.360 (0.758-2.442) | 0.303 |
| T3 | 6.055 (4.223-8.683) | <0.001 | 0.685 (0.492-1.095) | 0.114 |
| T4 | 33.880 (23.095-49.703) | <0.001 | 0.459 (0.284-0.744) | 0.002 |
| AJCC N classification§ |  |  |  |  |
| N0 | Reference | NA | Reference | NA |
| N1 | 1.907 (1.470-2.475) | <0.001 | 0.215 (0.154-0.299) | <0.001 |
| Multifocality |  |  |  |  |
| No | Reference | NA | Reference | NA |
| Yes | 1.243 (0.967-1.599) | 0.09 | 0.761 (0.557-1.039) | 0.085 |

DTC, Differentiated Thyroid Carcinoma, SBM, Synchronous Bone Metastases, OR, odds ratio, CI, Confidence Interval, NA, Not Applicable.

† Including American Indians, Alaska Natives and Asian-Pacific Islanders.

‡ Divorced, separated, single (never married), and widowed

§ According to the seventh edition of the AJCC Cancer Staging manual.
