# Supplementary material for: Incidence and Predictors of Synchronous Bone Metastasis in Newly Diagnosed Differentiated Thyroid Cancer: A Real-World Population-Based Study
Source: Front Surg. 2022 Jan 24;9:778303. doi: 10.3389/fsurg.2022.778303 (PMC8819693; doi:10.3389/fsurg.2022.778303)
Supplement: Supplementary Table S3 — Detailed scores of predictors in the nomograms. [file Table_3.DOCX]

Supplemental table 3 Detailed Scores of Predictors in the Nomograms

| Variable |  | Scores |
| --- | --- | --- |
| Age at diagnosis, Years |  |  |
| 18-44 |  | 0 |
| 45-54 |  | 35 |
| 55-64 |  | 65 |
| ≥65 |  | 80 |
| Race |  |  |
| White |  | 0 |
| Black |  | 32 |
| Others† |  | 18 |
| Gender |  |  |
| Male |  | 15 |
| Female |  | 0 |
| Histologic type |  |  |
| Papillary |  | 0 |
| Follicular |  | 79 |
| AJCC T classification§ |  |  |
| T1 |  | 0 |
| T2 |  | 28 |
| T3 |  | 44 |
| T4 |  | 100 |
| AJCC N classification§ |  |  |
| N0 |  | 0 |
| N1 |  | 21 |

including American Indians, Alaska Natives and Asian-Pacific Islanders.

† including American Indians, Alaska Natives and Asian-Pacific Islanders.

§ according to the seventh edition of the AJCC Cancer Staging manual.
