# Supplementary material for: Incidence and Predictors of Synchronous Bone Metastasis in Newly Diagnosed Differentiated Thyroid Cancer: A Real-World Population-Based Study
Source: Front Surg. 2022 Jan 24;9:778303. doi: 10.3389/fsurg.2022.778303 (PMC8819693; doi:10.3389/fsurg.2022.778303)
Supplement: Supplementary Table S4 — Univariable analysis for OS and CSS in patients with DTC diagnosed with SBM. [file Table_4.docx]

Supplemental table 4. Univariable Analysis for OS and CSS in Patients with DTC diagnosed with SBM

| Variable | Overall Survival | | Cancer-specific Survival | |
| --- | --- | --- | --- | --- |
|  | HR (95% CI) | p Value | HR (95% CI) | p Value |
| Year at diagnosis |  |  |  |  |
| 2010-2013 | Reference | NA | Reference | NA |
| 2014-2016 | 0.733 (0.437-1.230) | 0.240 | 0.616 (0.348-1.089) | 0.095 |
| Age at diagnosis, Years |  |  |  |  |
| 18-44 | Reference | NA | Reference | NA |
| 45-54 | 0.642 (0.215-1.916) | 0.427 | 0.471 (0.126-1.756) | 0.262 |
| 55-64 | 1.227 (0.497-3.028) | 0.657 | 1.352 (0.507-3.605) | 0.546 |
| ≥65 | 1.685 (0.718-3.954) | 0.230 | 1.657 (0.650-4.225) | 0.290 |
| Race |  |  |  |  |
| White | Reference | NA | Reference | NA |
| Black | 1.226 (0.669-2.247) | 0.510 | 0.978 (0.479-1.997) | 0.952 |
| Others† | 0.804 (0.430-1.502) | 0.493 | 0.704 (0.345-1.437) | 0.335 |
| Gender |  |  |  |  |
| Male | Reference | NA | Reference | NA |
| Female | 1.014 (0.654-1.574) | 0.950 | 1.122 (0.685-1.838) | 0.649 |
| Insurance situation |  |  |  |  |
| Insured | Reference | NA | Reference | NA |
| Uninsured | 1.577 (0.576-4.317) | 0.375 | 0.951 (0.233-3.890) | 0.945 |
| Marital status |  |  |  |  |
| Married | Reference | NA | Reference | NA |
| Unmarried‡ | 0.995 (0.976-1.014) | 0.589 | 0.981 (0.954-1.008) | 0.168 |
| Unknown | 1.010 (0.947-1.078) | 0.759 | 1.020 (0.957-1.088) | 0.539 |
| Histologic type |  |  |  |  |
| Papillary | Reference | NA | Reference | NA |
| Follicular | 0.884 (0.553-1.413) | 0.607 | 0.954 (0.570-1.595) | 0.856 |
| AJCC T classification§ |  |  |  |  |
| T1 | Reference | NA | Reference | NA |
| T2 | 0.952 (0.393-2.305) | 0.913 | 0.945 (0.330-2.692) | 0.912 |
| T3 | 1.016 (0.484-2.133) | 0.966 | 1.134 (0.476-2.702) | 0.776 |
| T4 | 3.020 (1.488-6.126) | 0.002 | 3.478 (1.533-7.889) | 0.003 |
| AJCC N classification§ |  |  |  |  |
| N0 | Reference | NA | Reference | NA |
| N1 | 1.904 (1.226-2.956) | 0.004 | 2.020 (1.240-3.290) | 0.005 |
| Metastatic sites≥2 |  |  |  |  |
| No | Reference | NA | Reference | NA |
| Yes | 4.014 (2.510-6.402) | <0.001 | 6.224 (3.512-11.028) | <0.001 |
| Multifocality |  |  |  |  |
| No | Reference | NA | Reference | NA |
| Yes | 0.903 (0.581-1.402) | 0.649 | 0.746 (0.454-1.220) | 0.242 |
| Surgery |  |  |  |  |
| No | Reference | NA | Reference | NA |
| Lobectomy | 1.101 (1.038-1.168) | 0.001 | 1.131 (1.058-1.209) | <0.001 |
| Subtotal or near-total thyroidectomy | 8.323 (2.679-25.856) | <0.001 | 8.391 (2.700-26.072) | <0.001 |
| Total thyroidectomy | 0.976 (0.955-0.998) | 0.035 | 0.984 (0.951-1.019) | 0.367 |
| Radiation therapy |  |  |  |  |
| No | Reference | NA | Reference | NA |
| Radiation beam or radioactive implants | 1.019 (0.992-1.047) | 0.167 | 1.039 (0.989-1.091) | 0.132 |
| Radioisotopes | 0.991 (0.964-1.019) | 0.528 | 1.018 (0.969-1.069) | 0.488 |
| Combination¶ | 0.996 (0.960-1.034) | 0.850 | 1.028 (0.973-1.085) | 0.330 |
| Chemotherapy |  |  |  |  |
| No | Reference | NA | Reference | NA |
| Yes | 3.756 (2.051-6.876) | <0.001 | 4.076 (2.156-7.706) | <0.001 |

DTC, Differentiated Thyroid Carcinoma, SBM, Synchronous Bone Metastases, HR, hazard ratio, CI, Confidence Interval, NA, Not Applicable.

† including American Indians, Alaska Natives and Asian-Pacific Islanders.

‡ divorced, separated, single (never married), and widowed

§ according to the seventh edition of the AJCC Cancer Staging manual.

¶ combination of beam with implants or isotopes
